# Association of visceral adipose tissue with albuminuria and interaction between visceral adiposity and diabetes on albuminuria

**DOI:** 10.1007/s00592-024-02271-8

**Published:** 2024-04-01

**Authors:** Yufang Liu, Dan Zhao, Sanbao Chai, Xiaomei Zhang

**Affiliations:** Department of Endocrinology, Peking University International Hospital, Beijing, 102206 People’s Republic of China

**Keywords:** Visceral fat, Diabetes, Albuminuria, Interaction

## Abstract

**Aims:**

To explore the correlation between visceral adipose tissue and albuminuria, and whether there is interaction between visceral adipose tissue and diabetes on albuminuria.

**Methods:**

The study subjects were adult subjects (age ≥ 18 years) from the National Health and Nutrition Examination Surveys (NHANES) database of the USA in 2017–2018. Visceral fat area (VFA) was measured by dual-energy X-ray absorptiometry (DXA). Subjects were divided into three groups according to VFA: low (VFA 0–60cm^2^), medium (VFA 60–120 cm^2^) and high (VFA ≥ 120 cm^2^). Albuminuria was defined as urinary albumin-to-creatinine ratio (UACR) ≥ 30 mg/g. The statistical analysis software used is STATA 17.0.

**Results:**

Data pertaining to 2965 participants (2706 without albuminuria) were included in the analysis. High VFA is an independent risk factor for albuminuria (OR 1.367, 95% CI 1.023–1.827). In the low-VFA group, there is no significant association between diabetes and albuminuria (OR 1.415, 95% CI 0.145–13.849). In the medium-VFA group, diabetes is an independent risk factor for albuminuria (OR 2.217, 95% CI 1.095–4.488). In the high-VFA group, diabetes is also an independent risk factor for albuminuria (OR 5.150, 95% CI 3.150–8.421). There is an additive interaction between high VFA (VFA ≥ 120 cm^2^) and diabetes on the effect of albuminuria (RERI 3.757, 95% CI 0.927–6.587, *p* = 0.009), while no multiplication interaction (OR 1.881, 95% CI 0.997–1.023, *p* = 0.141).

**Conclusions:**

High VFA may represent an independent risk factor for albuminuria. The amount of visceral fat may affect the effect of diabetes on albuminuria. The higher the visceral fat, the stronger the correlation between diabetes and albuminuria should be present. We suppose an additive interaction between VFA and diabetes on the effect of albuminuria.

## Introduction

As one of the common chronic diseases, diabetes is a global public health problem, which brings great challenges to human health. Diabetic nephropathy, one of the chronic microvascular complications of diabetes, causes a great impact on the expectancy and quality of life of diabetic patients. Albuminuria is one of the early manifestations of diabetic nephropathy. In the overall population, diabetes is an independent risk factor for albuminuria, which has been confirmed by numerous studies [1, 2].

Obesity is a condition characterized by insulin resistance, contributing to an elevated risk of type 2 diabetes mellitus [3]. Additionally, obesity plays a role in the progression of type 2 diabetes from the early stages of the disease [4]. Obesity and its metabolic complications also play an important role in the onset and progression of chronic kidney disease (CKD) [5, 6]. However, not all types of adipose tissue contribute equally to kidney injury. Individuals who predominantly store fat in the abdominal area (especially visceral area) tend to have higher risk of metabolic abnormality, such as type 2 diabetes and cardiovascular disease. It was demonstrated by recent studies that visceral adipose tissue (VAT) is more strongly associated with kidney injury [7–9], insulin resistance [10, 11] and cardiovascular disease [12] in several clinical conditions, compared with subcutaneous adipose tissue (SAT).

Previous studies have shown that both diabetes and VAT are closely related to albuminuria, however, few studies have focused on the interaction between diabetes and VAT in the risk of albuminuria. The aim of this study was to verify the association between VFA and albuminuria and to further explore the interaction between diabetes and high VFA in the risk of albuminuria.

### Methods

## Population

NHANES is a population-based, cross-sectional survey aimed to accomplish data collection about the health and nutrition of the U.S. residents. The survey was approved by the National Center for Health Statistics (NCHS) institutional review board, and all subjects signed written informed consent.

Data of subjects in the American NHANES 2017–2018 survey cycle were analyzed. NHANES 2017–2018 data are publicly available and can be accessed online (https://www.cdc.gov). Individuals lack relevant examinations or relevant data were excluded from the analyses. The analyses of the present study were limited to adult individuals, which means all participants were 18 years of age or older.

In the present study, respondents were categorized by ethnicity into white, black, Hispanic, Asian and other races. Body mass index (BMI) was calculated by dividing body weight in kg by square of height in meters. NHANES provides data for three consecutive blood pressure (BP) measurements and we used the second one to avoid deviation caused by emotional tension, physical activities or other factors that can make influence on blood pressure.

### Determination of visceral fat areas

In 2017–2018, whole body DXA scans were administered in the NHANES mobile examination center (MEC). The NHANES whole body scans through DXA provide nationally representative data on abdominal soft tissue composition and fat distribution of respondents. Visceral adipose tissue (VAT) were defined by the Hologic APEX software which was used in the scan analysis. Fat of visceral area inside abdominal cavity were measured at the approximate interspace location of L4 and L5 vertebra. Subjects were divided into three groups according to VFA: low (VFA 0 ~ 60cm^2^), medium (VFA 60 ~ 120 cm^2^) and high (VFA ≥ 120 cm^2^).

### Definition of albuminuria

Urinary albumin was measured using the latex agglutination method. Urinary albumin-to-creatinine ratio (UACR) was calculated by dividing the urinary albumin in milligram (mg) by urinary creatinine in gram. In the present study, albuminuria was defined as UACR ≥ 30 mg/g [13].

### Definition of diabetes

In the present study, individuals meeting any of the following conditions were diagnosed as diabetic patients: (1) confirmed history of diabetes diagnosis in questionnaire; (2) HbA1c level ≥ 6.5%; (3) fasting glucose level ≥ 7.0 mmol/L [14]. In NHANES, defining diabetes type through questionnaires or laboratory data is challenging. Therefore, we did not make a specific classification of diabetes patients or exclude those with type 1 diabetes in the study.

### Statistical analysis

The NHANES used a complex, multistage, probability sampling design to select participants representative of non-institutionalized US civilian, so we take this into account in our analyses by using sample weights to adjust for the unequal probability of selection into the survey and to adjust for the possible bias resulting from nonresponse according to NHANES analytic guidelines. The Kolmogorov–Smirnov method was performed to evaluate the data distribution. Continuous variables were represented as mean ± standard deviation (SD) for normally distributed data or medians (interquartile ranges, IQR) for abnormally distributed data. Chi-squared test, Mann–Whitney U-test or independent t-test was applied to evaluate the between-group differences when appropriate. Categorical variables were represented as frequency (percentage), and Chi-squared test was performed to compare the differences between groups. Logistic regression was performed to adjust for potential confounders when appropriate.

Logistic regression analysis was used to evaluated interactive effect between candidate risk factors on dependent variables on both a multiplicative scale and additive scales [15]. Relative excess risk due to the interaction (RERI) was used to evaluate the interaction based on the additive scale [16]. RERI is the excess risk due to the interaction relative to the risk without exposure, and it was interpreted no additive interaction between candidate variables if the 95% CI of RERI contained 0.

*p* Values < 0.05 was considered to be indicative of statistical significance. All statistical analyses were performed by using the STATA 17.0 software.

## Results

Baseline characteristics of the study population are summarized in Table 1. Data pertaining to 2965 participants (2706 without albuminuria) were included in the analysis. Individuals in the albuminuria group were significantly older and had higher BMI, VFA, systolic blood pressure (SBP), diastolic blood pressure, total triglyceride (TG), UA and HbA1c but lower serum creatinine and high-density lipoprotein cholesterol (HDL-c) than individuals without albuminuria (*p* < 0.05 for all). In the albuminuria group, the proportion of diabetes was higher (*p* < 0.001) as expected. There was no significant difference in the proportion of male and current smoker between the two groups.

**Table 1:** Characteristics of the study population by UACR

	UACR < 30 mg/g (*n* = 2706)	UACR ≥ 30 mg/g (*n* = 259)	*p*
Male (%)	1326(49%)	118(46%)	0.290
Age (years)	38.2 ± 12.5	43.0 ± 12.0	< 0.001
*Race (%)*			
White	839 (31%)	66 (25%)	0.304
Black	597 (22%)	63 (24%)	
Hispanic	668 (25%)	66 (25%)	
Asian	442 (16%)	43 (17%)	
Other	160 (6%)	21 (8%)	
Current smoker (%)	947 (35%)	101 (39%)	0.198
BMI (kg/m^2^)	29.3 ± 7.4	31.8 ± 8.2	< 0.001
VFA (cm^2^)	95.4 ± 54.0	121.0 ± 63.1	< 0.001
SBP (mmHg)	119 ± 16	131 ± 24	< 0.001
DBP (mmHg)	73 ± 12	77 ± 15	< 0.001
Serum creatinine (umol/L)	72.5 (61.4,84.9)	68.1 (56.6,83.1)	< 0.001
TG (mmol/L)	1.23 (0.84,1.84)	1.51 (1.04,2.51)	< 0.001
TC (mmol/L)	4.84 ± 1.00	4.93 ± 1.02	0.158
HDL-c (mmol/L)	1.36 ± 0.39	1.28 ± 0.42	0.004
UA (µmol/L)	316 ± 85	330 ± 105	0.019
Diabetes (%)	224 (8.6%)	82 (32%)	< 0.001
HbA1c (%)	5.6 ± 0.8	6.4 ± 1.9	< 0.001

*UACR* urinary albumin creatinine ratio, *BMI* body mass index, *VFA* visceral fat area, *SBP* systolic blood pressure, *DBP* diastolic blood pressure, *TG* triglyceride, *TC* total cholesterol, *HDL-c* high-density lipoprotein cholesterol, *UA* uric acid, *HbA1c* glycosylated hemoglobin

**Fig. 1:**
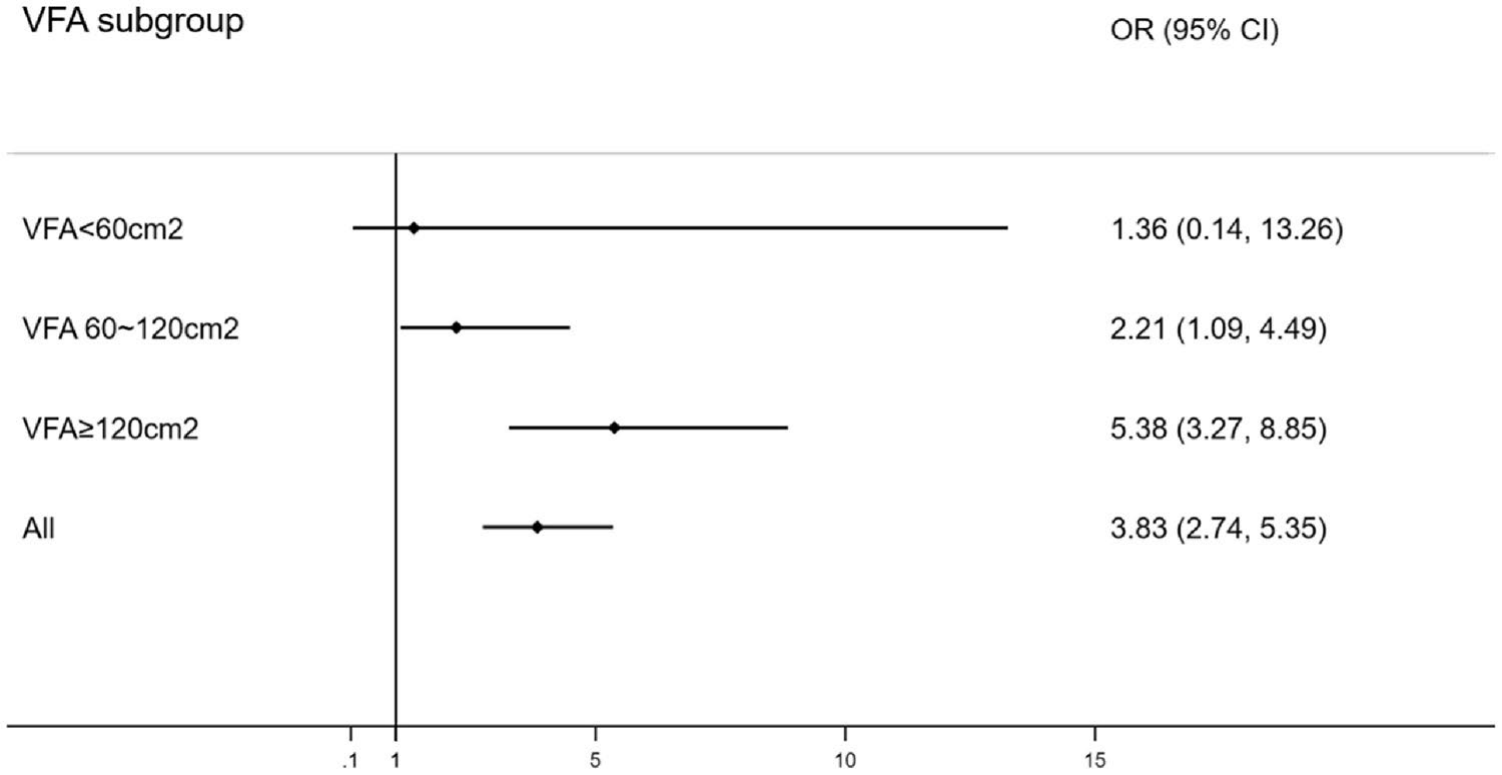
Forest plots of OR and 95% CI of diabetes on albuminuria, stratified by VFA

As shown in Table 2, after adjusting for confounding factors such as age, sex, race, smoking, and blood pressure, diabetes was an independent risk factor for albuminuria. But when we grouped all subjects according to visceral fat area, diabetes had a different effect on albuminuria among the different groups. In the high-VFA group, diabetes was an independent risk factor for albuminuria (OR 5.150, 95% CI 3.150–8.421, *p* < 0.001). In the medium-VFA group, diabetes was still an independent risk factor for albuminuria (OR 2.217, 95% CI 1.095–4.488, *p* < 0.001). While in the low-VFA group, there was no significant association between diabetes and albuminuria (OR 1.415, 95% CI 0.145–13.849, *p* = 0.765) (Fig. 1).

**Table 2:** Association of prevalence of albuminuria with diabetes, sorted by VFA

	VFA < 60cm^2^		VFA 60 ~ 120 cm^2^		VFA ≥ 120 cm^2^		All	
	*N* = 1859		*N* = 1382		*N* = 814		*N* = 4055	
	OR (95% CI)	*p*	OR (95% CI)	*p*	OR (95% CI)	*p*	OR (95% CI)	*p*
Model 1	2.014 (0.249, 16.301)	0.512	2.712 (1.395, 5.275)	0.003	4.903 (3.175, 7.570)	< 0.001	5.068 (3.767, 6.817)	< 0.001
Model 2	2.128 (0.255, 17.784)	0.486	2.263 (1.132, 4.526)	0.021	4.842 (3.061, 7.657)	< 0.001	4.272(3.115, 5.860)	< 0.001
Model 3	1.364 (0.140, 13.260)	0.789	2.214 (1.093, 4.485)	0.027	5.380 (3.271, 8.849)	< 0.001	3.834 (2.745, 5.353)	< 0.001

Model 1 was unadjusted (univariate). Model 2 was adjusted for age, sex and race. Model 3 was adjusted for model 2 adjustments plus smoking status, SBP and DBP

After adjusting for confounding factors such as age, sex, race, diabetes, smoking, and blood pressure, VFA was an independent risk factor for albuminuria. While in diabetes group, VFA was not associated with albuminuria (OR 1.702, 95% CI 0.971–2.983, *p* = 0.063). In non-diabetes group, VFA was not associated with albuminuria either (OR 1.259, 95% CI 0.888–1.786, *p* = 0.196) (Table 3).

**Table 3:** Association of prevalence of albuminuria with VFA (dm^2^), sorted by Glycemic Status

	Diabetes		Non-diabetes		All	
	*N* = 307		*N* = 2562		*N* = 2869	
	OR (95% CI)	*p*	OR (95% CI)	*p*	OR (95% CI)	*p*
Model 1	1.875 (1.181, 2.976)	0.008	1.436 (1.078, 1.914)	0.014	2.045 (1.648, 2.539)	< 0.001
Model 2	1.838 (1.098, 3.075)	0.021	1.486 (1.072, 2.062)	0.018	1.918 (1.490, 2.470)	< 0.001
Model 3	1.721 (0.978, 3.027)^§^	0.063	1.259 (0.888, 1.786)^*^	0.196	1.367 (1.023, 1.827)^#^	0.034

Model 1 was unadjusted (univariate). Model 2 was adjusted for age, sex, and race

^§^Model 3 was adjusted for model 2 adjustments plus smoking status, SBP, and duration of diabetes

*Model 3 was adjusted for model 2 adjustments plus smoking status and SBP

^#^Model 3 was adjusted for model 2 adjustments plus smoking status, SBP, and diabetes

Results of interaction analysis are shown in Table 4. Subjects were divided into four groups according to VFA and diabetes: low VFA (< 120 cm^2^) without diabetes, low VFA with diabetes, high VFA (≥ 120 cm^2^) without diabetes, high VFA with diabetes, with the first group as the reference group. Compared to the control group, individuals with high VFA but without diabetes have a higher risk of albuminuria (OR 1.516, 95% CI 1.038–2.216, *p* = 0.032) after adjusting for age, sex and race. Individuals with low VFA and diabetes have a higher risk of albuminuria (OR 2.315, 95% CI 1.217–4.404, *p* = 0.011). Individuals with high VFA and diabetes have the highest risk of albuminuria (OR 6.763, 95% CI 4.334–10.554, *p* < 0.001). Interaction analysis shows that there is an additive interaction between diabetes and VFA on albuminuria (RERI 3.757, 95% CI 0.927–6.587, *p* = 0.009), and there is no multiplicative interaction between diabetes and VFA on albuminuria (OR 1.881, 95% CI 0.997–1.023, *p* = 0.141).

**Table 4:** Interaction Between VFA and diabetes on presence of albuminuria

	VFA < 120 cm^2^	VFA ≥ 120 cm^2^	Additive interaction	Multiplicative interaction
	OR(95% CI)	OR(95% CI)	RERI(95% CI)	OR(95% CI)
	*p*	*p*	*p*	*p*
Diabetes (−)	1 (Ref.)	1.516 (1.038, 2.216) 0.032	3.757 (0.927, 6.587) 0.009	1.881 (0.997, 1.023) 0.141
Diabetes(+)	2.315 (1.217, 4.404) 0.011	6.763(4.334,10.554) < 0.001		

Adjusted for age, sex, race

*Ref.* reference

## Discussion

In low-VFA population, diabetes was not related to albuminuria, while in subjects with medium or high VFA, diabetes was an independent risk factor for albuminuria, consistent with conventional opinion [1, 2]. Among individuals with high VFA (VFA ≥ 120 cm^2^), diabetes had the strongest effect on albuminuria. The association of diabetes with albuminuria was discrepant in participants with different level of VFA, indicating that visceral fat and diabetes combined additively on pathophysiological changes of kidney. According to our results, diabetes may not be independent risk factor for albuminuria in people with low visceral fat, while there were sufficient evidences that albuminuria is a common complication of lean type 1 diabetes [1, 2]. There are some factors that may confound the present results, such as duration of the disease, treatment, and the presence of other complications (e.g. retinopathy). More studies are needed to confirm or disprove these findings in the future.

It is interesting that similar findings appeared in an Asian study. Bouchi and colleagues found there was a stronger association between blood pressure and albuminuria in individuals with high visceral adiposity than those with low visceral adiposity (*β* 0.263, *p* = 0.001 versus *β* 0.140, *p* = 0.080). Authors made an inference that visceral adiposity could modify the association of blood pressure with albuminuria [17]. Based on the findings of the above study and our study, we can speculate that visceral fat is involved in regulating the association of multiple metabolic factors with kidney outcomes.

Conventionally we used BMI to assess adiposity, while there are some noteworthy limitations for BMI to assess adiposity. The value of central adiposity measurement is highlighted in studies showing that patients with low or normal BMI but elevated waist circumference, also known as abdominal obesity or sarcopenic obesity, are at the highest risk of end-stage renal disease (ESRD) and death [18–20]. Studies have demonstrated that visceral adipose tissue is a major contributor to cardiovascular disease and kidney function decline [21–23]. Conversely, fat storage preferential in the lower body depot may serve as a metabolic buffer, protecting other tissues from lipotoxicity [21]. Thereby in the present study, we chose VFA as adiposity metric to probe into the effect of specific type of obesity, that is visceral fat accumulation on albuminuria and its interaction with diabetes.

Previous studies have suggested that visceral fat accumulation evaluating by cumulative average visceral adiposity index (VAI) is independently associated with increased urinary albumin excretion [24, 25]. Most recently, Zhou et al. conducted a study in 10,132 participants from China, which showed that higher cumulative average VAI was associated with a higher risk of progression of renal disease in patients with type 2 diabetes mellitus [26]. The VAI used in these previous studies to quantify visceral fat was calculated indirectly, using a formula that included BMI, waist circumference and other parameters, rather than directly measuring it. Research of Japanese scholars revealed that VFA by the bioelectrical impedance analysis method is also associated with an increase in UACR [27]. In this study, DXA was used to quantify visceral fat more directly and accurately, further verifying the conclusions of previous studies, that high visceral fat is a risk factor for adverse kidney outcomes.

There are several mechanisms that can explain the negative influence of visceral adiposity on kidney. In one aspect, non-specific inflammatory responses are regarded as key mechanism involved in central obesity-related target organ manifestations [28]. The dysregulated adipocytes and macrophages within visceral fat component are metabolically active and may secrete or regulate various adipokines and proinflammatory cytokines, such as leptin [29], adiponectin [30], resistin [31], visfatin [32, 33], and vascular endothelial growth factor [34, 35], which may lead to low-grade systemic inflammation, insulin resistance, dyslipidemia, and/or increased synthesis of vasoactive and fibrogenic substances, eventually leading to impairment of vascular endothelial cells and kidney function [36–40]. On the other hand, the excess visceral adipose tissue accumulated in and around the kidneys, may result in renal physical compression and raise intrarenal tissue pressure, which increase renal sympathetic nervous system (SNS) activity and sequentially activate the renin–angiotensin–aldosterone system (RAAS) [41, 42] and mineralocorticoid receptor [43]. Moreover, renal sinus fat accumulation is associated with glomerular hypertrophy and angiotensinogen produced in the renal tissue. Intratubular angiotensinogen stimulates the production of angiotensin II, which elevates blood pressure by acting on angiotensin II type 1 receptors [44]. These would combinedly cause gradual loss of nephrons [45].

VFA is closely associated with albuminuria in the whole population. In contrast, our subgroup analysis revealed that VFA was not associated with increased adjusted risk of albuminuria in participants with or without diabetes. These data emphasize the complex interplay between diabetes and obesity in the modulation of albuminuria.

We present an interaction analysis of diabetes with VFA on albuminuria in a large cohort stratified by baseline diabetes status and VFA category. Albuminuria was more common as VFA rose, but in the presence of diabetes, event rates were much greater. Indeed, participants with diabetes and low-VFA experienced higher adjusted event rates than high-VFA participants without diabetes. Eventually, our interaction analysis revealed a significant additive interaction between high visceral fat and diabetes with an increased risk of albuminuria. Even at an earlier stage, such interaction may be present. Previous study suggested a significant interaction between central obesity-related abnormal lipid metabolism and prediabetes, which exert a synergistic effect on microalbuminuria [46].

To the best of our knowledge, this is the first study trying to explore the interaction between VFA and diabetes on albuminuria. Moreover, not exhaustive enough but reliable database from NHANES and a relatively large sample size are strengths of this study.

However, we must also acknowledge limitations. First of all, we did not exclude those with type 1 diabetes in the study. Given the different pathogenesis of type 1 diabetes and type 2 diabetes, this may have some impact on the findings to a certain extent. Second, our work is observational cross-sectional study, so we could not make causality inference. Third, NHANES only report whether taking prescription for hypertension, but no specific types of antihypertensive agents (RAAS inhibitors, CCB, et al.), so we can’t eliminate the impact of RAAS inhibitors on the results. Moreover, participants were recruited before widespread use of sodium–glucose cotransporter 2 (SGLT-2) inhibitors or mineralocorticoid receptor antagonist (finerenone), which improve renal outcomes. Therefore, event rates in participants with diabetes may be higher in our study than contemporary data. Fourth, in clinical practice, we conduct three UACR tests to diagnose or exclude albuminuria, while in NHANES, subjects undergo a one-time examination. Fifth, there is a bias of abdominal VAT measures of DXA compared with MRI, but the high rank correlation makes DXA a good alternative to MRI which is more complicated and time-consuming [47]. Hopefully, there will be studies verifying our findings through MRI in the future. Last but not least, adiposity estimates of DXA were often sex and race/ethnicity specific [48], future studies may conduct subgroup analysis according to specific estimates of sex and race, or validate our findings through broader populations.

## Conclusions

High VFA may constitute an independent risk factor for albuminuria. The quantity of visceral fat could influence the impact of diabetes on albuminuria. The greater the visceral fat, the more pronounced the correlation between diabetes and albuminuria is expected to be. We suppose an additive interaction between VFA and diabetes on the effect of albuminuria.
